# Real-World Effectiveness of Targeted-Release Budesonide in IgA Nephropathy with Near-Universal SGLT2 Inhibitor Use

**DOI:** 10.1159/ajn/ablag015

**Published:** 2026-10-15

**Authors:** Thomas McDonnell, William R. Marshall, Sharmilee Rengarajan, Jude Allen, Rajkumar Chinnadurai, Smeeta Sinha, Philip A. Kalra

**Affiliations:** a Renal Department, Northern Care Alliance NHS Foundation Trust, Salford, UK; b Division of Cardiovascular Sciences, Faculty of Biology Medicine and Health, University of Manchester, Manchester, UK

**Keywords:** IgA nephropathy, Targeted-release formulation budesonide, Proteinuria

## Abstract

**Introduction:**

IgA nephropathy (IgAN) is the most common primary glomerulonephritis worldwide and a leading cause of end-stage kidney disease. Targeted-release formulation (TRF) budesonide demonstrated significant reductions in proteinuria and attenuation of estimated glomerular filtration rate (eGFR) decline in the randomised NefIgArd trial. However, real-world effectiveness and tolerability in routine clinical practice remain less well characterised. We report real-world outcomes following initiation of TRF budesonide in a tertiary renal centre.

**Methods:**

We conducted a retrospective cohort study of all patients with biopsy-proven IgAN initiated on TRF budesonide between 1st January 2024 and 31st July 2025 at a single renal centre in the UK, with follow-up until 31st January 2026. Outcomes were changes in urine protein-to-creatinine ratio (UPCR) and eGFR from baseline to 3, 6, and 9 months and to the latest follow-up, assessed within patients using the Wilcoxon signed rank test. Intention-to-treat (ITT; all 33 patients commencing therapy) and per-protocol (PP; ≥6 months of therapy and not switched directly from modified-release budesonide) analyses were performed. Pre- and post-treatment eGFR slopes were compared using linear mixed-effects models, and a historical comparator cohort matched on baseline UPCR was drawn from the Salford Kidney Study; both are reported as exploratory.

**Results:**

Thirty-three patients commenced TRF budesonide, of whom 23 met PP criteria. Median age was 44 (IQR 37–60) years, baseline UPCR 212 (133–289) mg/mmol, and eGFR 48 (30–61) mL/min/1.73 m^2^. Renin-angiotensin system (RAS) inhibitor and sodium-glucose cotransporter-2 (SGLT2) inhibitor use were 94% and 91%, respectively. At the latest follow-up (median 11.2 months after treatment initiation), UPCR decreased by 60% (IQR 31–71%) in the ITT cohort (*p* < 0.001) and 57% (IQR 26–72%) in the PP cohort (*p* < 0.001). eGFR was stable across the treatment period, with no significant change from baseline at 9 months or at the latest follow-up in either analysis set. Pre- to post-treatment slope comparison was confounded by initiation of SGLT2 inhibitor therapy within the pre-treatment observation window and is reported as exploratory. An adverse effect was recorded in 9 of 33 patients (27%). Seven patients (21%) discontinued treatment, 6 for non-serious adverse effects and 1 for a serious adverse event (staphylococcal septicaemia requiring hospital admission); treatment was temporarily suspended in 1 further patient.

**Conclusion:**

In a real-world IgAN cohort with near-universal RAS and SGLT2 inhibitor use, TRF budesonide was associated with substantial proteinuria reduction, additive to optimised background therapy. eGFR was stable over the treatment period. Slope-based analyses could not be interpreted, because background SGLT2 inhibitor therapy was initiated within the pre-treatment observation window in nearly every patient. Discontinuation for adverse effects was more frequent than in trials, though symptoms were generally mild; adverse event frequencies are not directly comparable given retrospective ascertainment.

## Introduction

IgA nephropathy (IgAN) is the most prevalent primary glomerulonephritis worldwide, accounting for approximately 30–40% of primary glomerular disease on biopsy in adults [1, 2]. The pathophysiological cornerstone of IgAN is mucosal overproduction of galactose-deficient IgA1 (Gd-IgA1); this triggers the formation of circulating immune complexes that deposit in the glomerular mesangium, activating complement and driving glomerulosclerosis and progressive CKD [3]. Proteinuria, a consequence of mesangial IgA deposition and resultant glomerular inflammation, is the best validated modifiable predictor of kidney disease progression in IgAN, and its reduction remains the primary therapeutic target [4].

The mucosal immune axis, particularly gut-associated lymphoid tissue, is central to the Gd-IgA1 glycosylation that characterises IgAN. This mechanism underpins the development of targeted-release formulation (TRF) budesonide, an enteric-coated formulation designed for pH-dependent release in the distal ileum and proximal colon, the site of Peyer’s patches, where IgA1-producing B-cell priming is concentrated [5]. By delivering high local budesonide concentrations at this mucosal site, the formulation aims to suppress Gd-IgA1 production while avoiding the well-recognised toxicities of systemic corticosteroids.

The phase III NefIgArd trial demonstrated that 9 months of TRF budesonide (16 mg/day) significantly reduced proteinuria, with a 27% decrease in UPCR at 9 months compared with placebo. In the full 2-year analysis, the time-weighted average eGFR over 2 years was 5.05 mL/min/1.73 m^2^ higher with TRF budesonide than placebo (95% CI: 3.24–7.38; *p* < 0.0001), corresponding to a difference in 2-year eGFR total slope of 2.78 mL/min/1.73 m^2^/year (95% CI: 1.39–4.17) by linear spline mixed-effects modelling, with estimates ranging from 1.82 to 2.95 mL/min/1.73 m^2^/year across the three analysis methods reported [6, 7]. These findings supported regulatory approval in both the USA and Europe. However, several features of the NefIgArd trial population limit direct translation into contemporary clinical practice: no participant was receiving a sodium-glucose cotransporter-2 (SGLT2) inhibitor at randomisation, only 5% commenced one during the trial, and the duration from biopsy to enrolment was substantially longer than is typical in UK clinical practice. Given the rapid and widespread adoption of SGLT2 inhibitors as standard-of-care background therapy for proteinuric CKD [8–10], the additive efficacy of TRF budesonide on top of near-universal SGLT2 inhibitor use is of central clinical importance.

Equally, the tolerability profile of TRF budesonide in routine clinical practice remains incompletely characterised. The NefIgArd trial population was subject to eligibility criteria that excluded patients at higher risk of corticosteroid-related harm, and the intensive monitoring inherent to trial participation may not reflect the adverse event burden encountered in routine nephrology practice.

Real-world evidence from patients treated outside trial conditions is therefore critical to contextualise the risk-benefit profile and to inform prescribing decisions. We report outcomes from a retrospective single-centre cohort study of all patients with biopsy-proven IgAN initiated on TRF budesonide at a tertiary UK renal centre. We characterise proteinuria response, eGFR trajectory, and adverse event profile.

## Methods

### Study Design and Setting

We conducted a retrospective cohort study of all patients with biopsy-proven IgAN who commenced TRF budesonide 16 mg daily at a tertiary renal centre (Salford Royal Hospital) in the UK between 1 January 2024 and 31 July 2025. Our centre serves a socioeconomically diverse population with a catchment area of 1.6 million. Patients were identified from pharmacy dispensing records, and clinical data were extracted from electronic health records.

### Patient Population

Eligible patients had a histopathological diagnosis of IgAN confirmed on kidney biopsy. No exclusion criteria were applied: all patients commencing TRF budesonide during the study period were included, and all contributed at least one post-treatment UPCR and eGFR measurement.

Treatment followed the licensed dosing regimen: TRF budesonide 16 mg daily in the morning at least 30 min before breakfast for 9 months, followed by a 1-month taper in most patients. All prescriptions were issued at the discretion of seven treating nephrologists with subspecialist interest in glomerulonephritis, with availability of local multidisciplinary review, consistent with national prescribing guidance.

### Data Collection

Baseline demographic and clinical data extracted included age, sex, ethnicity, time from diagnostic biopsy to treatment initiation, blood pressure, concomitant medications (renin-angiotensin system [RAS] inhibitors and SGLT2 inhibitors), prior corticosteroid exposure, and Oxford MEST-C classification scores where recorded in the diagnostic biopsy report. UPCR and eGFR (MDRD equation as reported in hospital laboratories [11]) were collected at baseline and at 3, 6, and 9 months after treatment initiation, and at the most recent available time point (designated “latest”). Pre-treatment eGFR data were collected from clinic records up to 5 years prior to treatment initiation. Adverse events and reasons for treatment discontinuation were systematically recorded from clinical letters and electronic records.

### Statistical Analysis

Continuous variables are presented as median (interquartile range [IQR]). Longitudinal changes in UPCR and eGFR are expressed as percentage change from baseline. All analyses were available at each timepoint, with no imputation for missing values. SGLT2 inhibitor exposure was ascertained at the index date (treatment initiation for the budesonide cohort, study enrolment for comparators); dates of SGLT2 inhibitor initiation were retrieved from the prescribing record to permit assessment of exposure timing relative to the pre-treatment observation window.

Differences between baseline and 9-month or latest values were assessed using the Wilcoxon signed rank test and expressed as median percentage change from baseline. Normality was not formally tested; non-parametric methods were applied to all within-patient comparisons given the skewed distribution of proteinuria and the small sample size.

eGFR trajectories were characterised using linear mixed-effects models with a fixed effect for time and patient-level random intercepts and random slopes, fitted separately to the 5 years before treatment initiation and to the period from initiation to latest follow-up. Population-level slopes were taken from the fixed effect for time and reported in mL/min/1.73 m^2^/year with Wald 95% confidence intervals. Individual-level slopes were estimated only where at least two measurements on distinct dates were available.

Analyses were conducted under two complementary frameworks. The intention-to-treat (ITT) analysis included all 33 patients who commenced TRF budesonide regardless of treatment duration or formulation switch history. The per-protocol (PP) analysis was restricted to the 23 patients who received ≥6 months of TRF budesonide and had not been switched directly from modified-release budesonide (Entocort EC), to avoid confounding from contiguous exposure to a different budesonide formulation. Two patients with earlier, non-contiguous modified-release budesonide exposure were retained.

### UPCR-Matched Comparator Cohort

Historical comparator patients were identified from the Salford Kidney Study (SKS) [12], comprising patients with biopsy-proven IgAN who had not received immunosuppressive therapy. The index date was treatment initiation for the TRF budesonide cohort and SKS enrolment for comparators; enrolment rather than date of IgA nephropathy diagnosis was used to minimise immortal time bias. Comparators were matched 1:1 by nearest neighbour on baseline UPCR, which accounts for a substantial proportion of the variation in subsequent eGFR trajectory in IgA nephropathy and was judged the covariate on which balance mattered most given the limited comparator pool. Multivariable propensity score matching was subsequently performed, with scores estimated from a logistic model including age, sex, baseline eGFR and log baseline UPCR, using 1:1 nearest-neighbour matching without replacement and a calliper of 0.20 standard deviations of the logit propensity score. A further specification adding exact matching on SGLT2 inhibitor status at index was attempted with callipers of 0.20, 0.10 and 0.05 standard deviations. Balance after matching was assessed for each specification using standardised mean differences (SMDs), with an SMD of >0.10 taken to indicate residual imbalance.

For comparator patients, eGFR measurements up to 2 years after the index date were extracted. Post-index eGFR slopes were estimated for each patient with at least two measurements on distinct dates, summarised as medians, and compared between groups using the Mann-Whitney U test. Baseline categorical variables were compared using Fisher’s exact test given the small cell counts.

As a further sensitivity analysis to avoid the loss of unmatched comparators, linear mixed-effects models with a treatment-by-time interaction were fitted to all post-index eGFR measurements over a common 3-year horizon in the PP cohort and in the comparators with at least two measurements on distinct dates. The interaction was adjusted sequentially for age, baseline eGFR, log-transformed baseline UPCR and SGLT2 inhibitor use at index.

## Results

### Cohort Characteristics

Between 1 January 2024 and 31 July 2025, 33 patients with biopsy-proven IgAN were initiated on targeted-release budesonide (ITT cohort). Of these, 3 had been switched directly from modified-release budesonide (Entocort EC), and 7 had been taking TRF budesonide for <6 months at the time of data entry, meaning 23 met PP criteria. Two patients had earlier, non-contiguous modified-release budesonide exposure and were retained in the PP cohort. Baseline characteristics are summarised in Table 1, alongside corresponding data from the NefIgArd trial TRF budesonide arm for contextual comparison.

**Table 1.** Baseline demographic and clinical characteristics of the real-world cohort compared with the NefIgArd trial Nefecon arm

Characteristic	Real-world cohort (*N* = 33)	NefIgArd trial – Nefecon arm (*N* = 182)
Age, years	44 (37–60)	43 (36–50)
Male sex, *n* (%)	18 (55)	117 (64)
White ethnicity, *n* (%)	23 (70)	138 (76)
Time from biopsy, months	14 (3–34)	29 (7–83)
Previous corticosteroid exposure, *n* (%)^a^	7 (21)	15 (8)
Oxford MEST-C score available, *n* (%)	28 (85)	Not reported
M1, *n* (%)^b^	22 (79)	Not reported
E1, *n* (%)^b^	11 (39)	Not reported
S1, *n* (%)^b^	23 (82)	Not reported
T1 or T2, *n* (%)^b^	9 (32)	Not reported
Any previous MR budesonide, *n* (%)^c^	5 (15)	Not reported
Switched directly from MR budesonide, *n* (%)^c^	3 (9)	Not reported
Systolic BP, mm Hg	130 (123–140)	126 (121–132)
Diastolic BP, mm Hg	84 (80–89)	79 (76–84)
Diabetes mellitus, *n* (%)	1 (3)	16 (9)
RAS inhibitor use at index date, *n* (%)	31 (94)	179 (98)
SGLT2 inhibitor use at index date, *n* (%)	30 (91)	0 (0)^d^
Baseline UPCR, mg/mmol	212 (133–289)	145 (102–199)^e^
Baseline eGFR, mL/min/1.73 m^2^	48 (30–61)	56 (46–71)

Continuous variables are presented as median (IQR) and categorical variables as *n* (%). The index date is the date of treatment initiation. eGFR in the real-world cohort was derived using the MDRD equation as reported by the hospital laboratory, whereas NefIgArd used the CKD-EPI equation.

BP, blood pressure; eGFR, estimated glomerular filtration rate; MR, modified release; RAS, renin-angiotensin system; SGLT2, sodium-glucose cotransporter-2; UPCR, urine protein-to-creatinine ratio.

^a^ In NefIgArd, systemic glucocorticoid or immunosuppressant treatment more than 12 months before randomisation; any within 12 months was exclusionary. The real-world figure is any documented prior corticosteroid exposure and is not directly comparable.

^b^ MEST-C percentages are of the 28 patients with an available score; the crescent (C) component was reported in only 7 biopsies (C1 in 3).

^c^ The 3 patients who switched directly from modified-release budesonide were excluded from the per-protocol analysis; the remaining 2 had earlier, non-contiguous exposure and were retained.

^d^ No NefIgArd participant was receiving an SGLT2 inhibitor at randomisation; 10 (5%) commenced one during the trial.

^e^ Converted from g/g to mg/mmol using the factor 1 g/g = 113 mg/mmol; original NefIgArd values 1.28 (0.90–1.76) g/g. NefIgArd measured UPCR on 24-h urine collection, whereas the real-world cohort used spot samples.

Median age was 44 (IQR 37–60) years; 55% were male, and 70% were of White ethnicity. Median time from biopsy to treatment initiation was 14 (IQR 3–34) months, substantially shorter than in NefIgArd (median 29 months). Prior corticosteroid exposure was documented in 21% of patients. Background therapy was highly optimised: baseline RAS inhibitor use was 94%, and SGLT2 inhibitor use was 91%, in contrast to the NefIgArd trial, in which no participant was receiving an SGLT2 inhibitor at randomisation, and only 5% commenced one during the trial. Median blood pressure at treatment initiation was 130/84 mm Hg. Baseline UPCR was 212 (IQR 133–289) mg/mmol and median eGFR was 48 (IQR 30–61) mL/min/1.73 m^2^. The median number of pre-treatment eGFR measurements per participant was 16 (IQR 10–19; range 3–29) and the median number of post-treatment eGFR measurements was 6 (IQR 4–8; range 2–22).

Oxford classification scores were available from biopsy reports for 28 patients (85%). Mesangial hypercellularity (M1) was present in 22 (79%) and segmental sclerosis (S1) in 23 (82%), while endocapillary hypercellularity (E1) was present in 11 (39%). Tubular atrophy and interstitial fibrosis were absent or minimal in the majority (T0 in 19 patients, 68%), with T1 in 8 patients (29%) and T2 in 1 patient (4%). Crescents were formally scored in 7 biopsies, of which 3 were C1.

### Proteinuria Response

Proteinuria responses are summarised in Table 2 and Figure 1a, b. In the ITT cohort, UPCR decreased by a median of 41% (IQR 7–71%) at 9 months (*p* = 0.002) and by 60% (IQR 31–71%) at latest follow-up (*p* < 0.001; median follow-up to latest measure 11.2 months after treatment initiation). In the PP cohort, UPCR reduction at latest follow-up was 57% (IQR 26–72%, *p* < 0.001). All 33 patients in the ITT cohort and all 23 in the PP cohort contributed a value at the latest timepoint; at 9 months, 28 patients contributed a UPCR and 27 an eGFR measurement.

**Table 2.** Efficacy outcomes: changes in UPCR and eGFR in intention-to-treat (ITT) and per-protocol (PP) analyses, with exploratory eGFR slope estimates

Outcome	ITT (*N* = 33)	PP (*N* = 23)
ΔUPCR at 9 months, % (IQR)	−41 (−71 to −7); *p* = 0.002	−44 (−73 to 14); *p* = 0.017
ΔUPCR at latest follow-up, % (IQR)	−60 (−71 to −31); *p* < 0.001	−57 (−72 to −26); *p* < 0.001
ΔeGFR at 9 months, % (IQR)	−4 (−21 to 16); *p* = 0.718	−4 (−21 to 15); *p* = 0.498
ΔeGFR at latest follow-up, % (IQR)	−1 (−14 to 11); *p* = 0.382	−4 (−13 to 9); *p* = 0.357
Pre-treatment eGFR slope, mL/min/1.73 m^2^/year (95% CI)	−2.74 (−4.13 to −1.34)	−2.75 (−4.43 to −1.07)
Post-treatment eGFR slope, mL/min/1.73 m^2^/year (95% CI)	−0.96 (−5.06 to 3.15)	−1.70 (−3.62 to 2.14)
Difference in slope, mL/min/1.73 m^2^/year (95% CI)	1.78 (−2.56 to 6.12); *p* = 0.423	1.05 (−4.15 to 6.25); *p* = 0.691
Median follow-up to latest measure, months	11.2 (7.0 to 13.3)	11.2 (7.0 to 13.3)

Values are median (IQR) unless otherwise stated. Changes from baseline were compared using the Wilcoxon signed-rank test; negative values denote a decrease from baseline. Analyses are available-case with no imputation, and the number of patients contributing at each timepoint is shown in Figures 1 and 2. eGFR was derived using the MDRD equation as reported by the hospital laboratory.

CI, confidence interval; eGFR, estimated glomerular filtration rate; ITT, intention-to-treat; IQR, interquartile range; PP, per protocol; UPCR, urine protein-to-creatinine ratio.

**Fig. 1.**
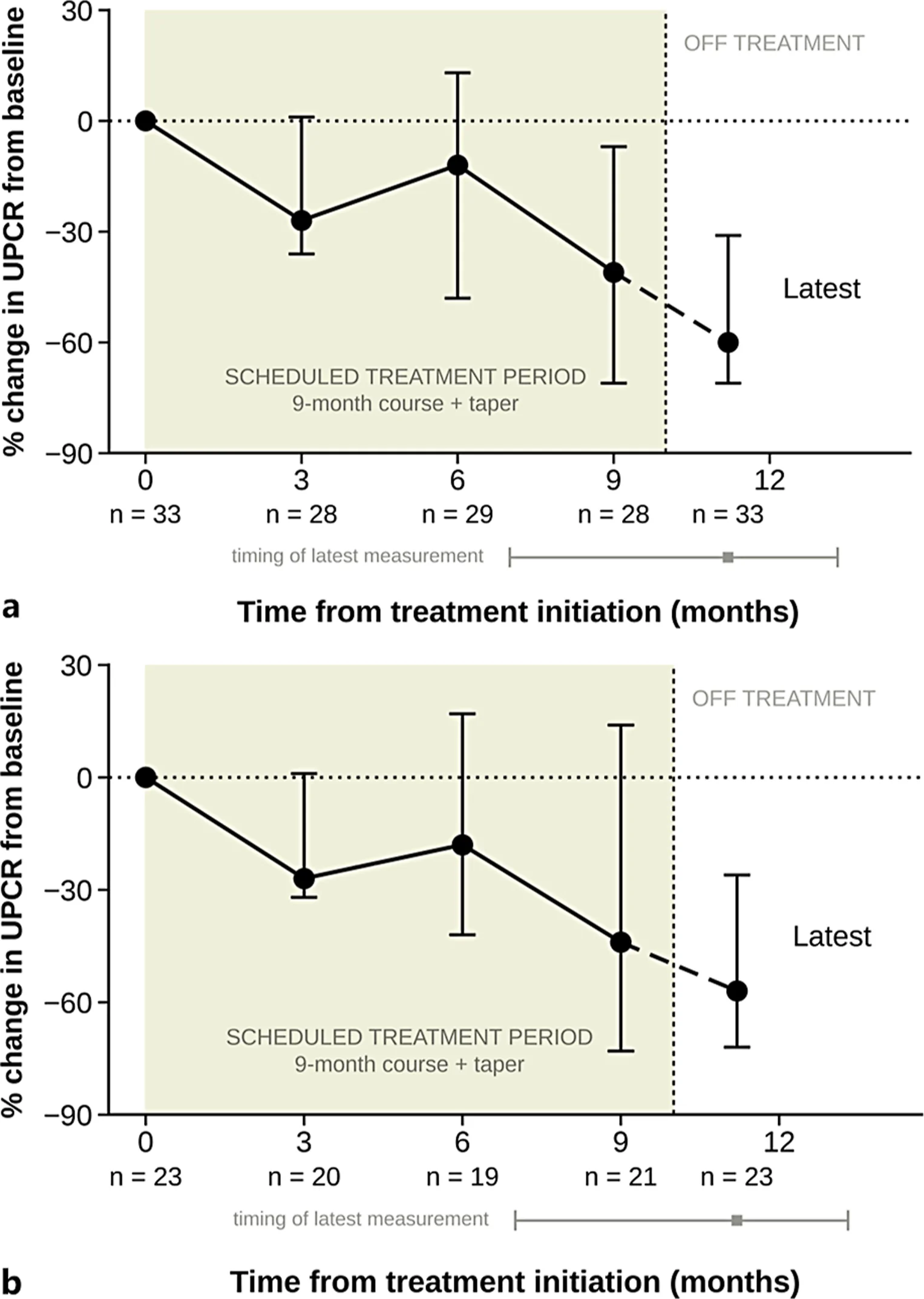
Median percentage change in UPCR from baseline following targeted-release budesonide, in the ITT cohort (**a**) and the PP cohort (**b**). Points are medians and vertical bars are the IQR; the baseline point is zero by definition. Negative values denote a reduction from baseline. The shaded region indicates the scheduled 9-month course and 1-month taper; actual exposure was shorter in patients who discontinued early. The final point is the latest available measurement, plotted at its median time from treatment initiation (11.2 months) rather than at a fixed visit; the dashed segment marks this interval as unscheduled. The grey bar beneath the axis shows the median and interquartile range of the timing of that measurement (IQR 7.0–13.3 months), so in a minority of patients it was recorded during rather than after treatment. The number of patients contributing at each timepoint is shown beneath the *x*-axis.

Of the ITT group, 28 of the 33 had a UPCR ≥100 mg/mmol at baseline. Of these, at latest follow-up, 23 (82%) and 8 (29%) achieved a UPCR of <100 mg/mmol and <50 mg/mmol, respectively. At the latest follow-up, 25 of 33 patients (76%) achieved at least a 30% reduction in UPCR from baseline, and 21 (64%) at least a 50% reduction.

### eGFR Trajectory

eGFR changes are summarised in Table 2 and Figure 2a, b. The change in eGFR from baseline to 9 months and to latest follow-up did not reach statistical significance in either the ITT (4% decrease at 9 months, *p* = 0.718; 1% at latest follow-up, *p* = 0.382) or PP analysis (4% decrease at 9 months, *p* = 0.498; 4% at latest follow-up, *p* = 0.357).

**Fig. 2.**
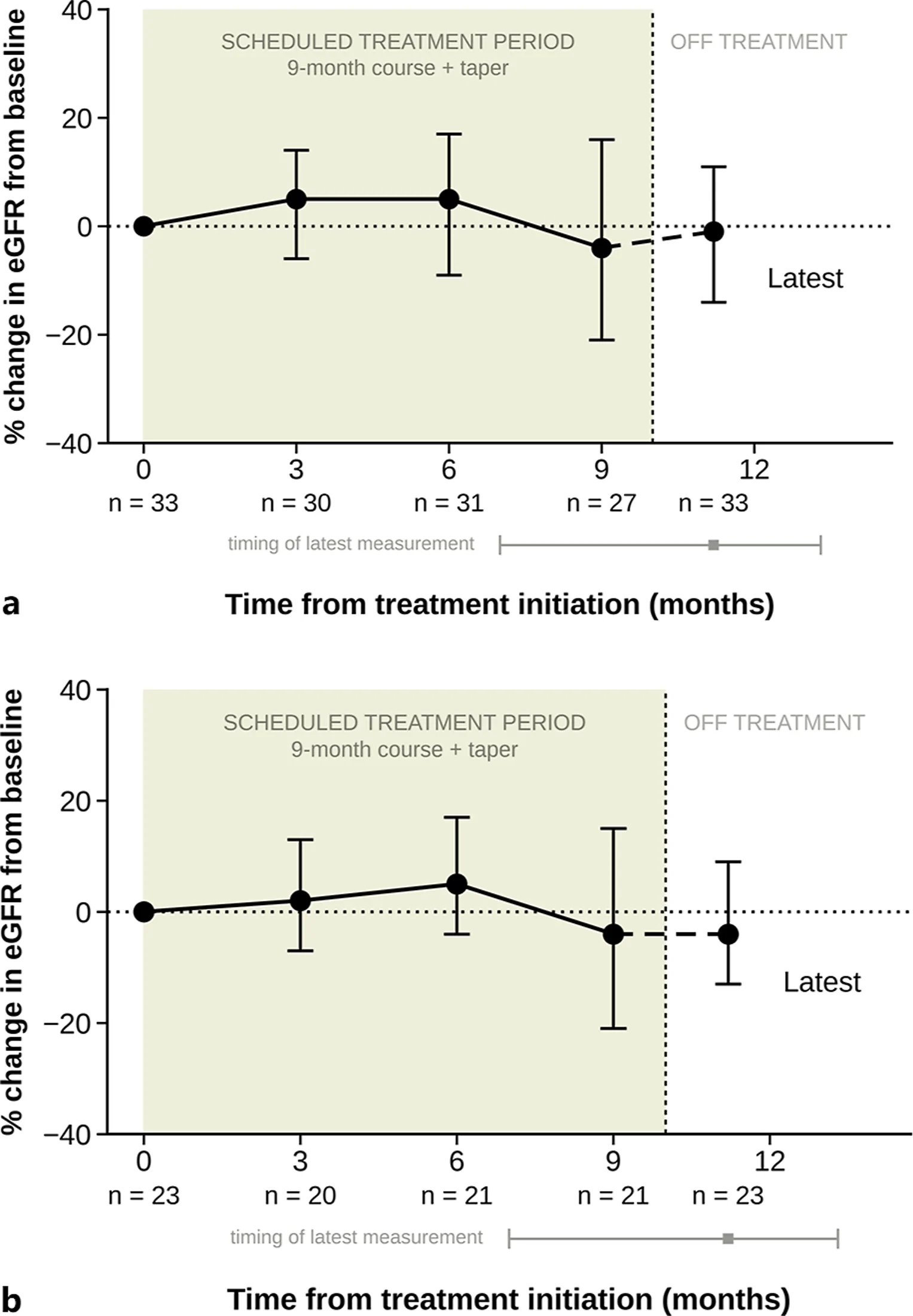
Median percentage change in eGFR from baseline following targeted-release budesonide, in the ITT cohort (**a**) and the PP cohort (**b**). Points are medians and vertical bars are the IQR; the baseline point is zero by definition. Negative values denote a decline from baseline. The shaded region indicates the scheduled 9-month course and 1-month taper; actual exposure was shorter in patients who discontinued early. The final point is the latest available measurement, plotted at its median time from treatment initiation (11.2 months) rather than at a fixed visit; the dashed segment marks this interval as unscheduled. The grey bar beneath the axis shows the median and interquartile range of the timing of that measurement (IQR 7.0–13.3 months), so in a minority of patients it was recorded during rather than after treatment. The number of patients contributing at each timepoint is shown beneath the *x*-axis.

Estimates of eGFR slope demonstrated a numerical improvement following treatment initiation. In the ITT cohort, the pre-treatment eGFR slope was −2.74 mL/min/1.73 m^2^/year (95% CI: −4.13 to −1.34), derived from a median of 3.4 years of pre-treatment observations. Following treatment initiation, the estimated post-treatment slope was −0.96 mL/min/1.73 m^2^/year (95% CI: −5.06 to 3.15), representing an attenuation of decline of 1.78 mL/min/1.73 m^2^/year (95% CI: −2.56 to 6.12; *p* = 0.423). A similar numerical and non-significant attenuation was observed in the PP analysis (1.05 mL/min/1.73 m^2^/year, 95% CI: −4.15 to 6.25; *p* = 0.691).

Dates of SGLT2 inhibitor initiation were recorded for 28 patients. SGLT2 inhibitor therapy preceded budesonide initiation in all 28, by a median of 16.9 months (IQR 7.8–28.7), and by at least 6 months in 23. In 27 of these, initiation fell within the pre-treatment observation window rather than before it, at a median of 1.5 years into that window, with start dates between 2021 and 2024. In an exploratory within-patient analysis estimating trajectory either side of the SGLT2 inhibitor start date, the median pre-treatment slope was −1.79 mL/min/1.73 m^2^/year before initiation (24 patients, median span 1.46 years) and −5.95 mL/min/1.73 m^2^/year after initiation but before budesonide (23 patients, median span 1.09 years) (online suppl. Table S5; for all online suppl. material, see https://doi.org/10.1159/ajn/ablag015). Among the 19 patients in whom both trajectories were estimable, decline was steeper after SGLT2i initiation by a median of 2.40 mL/min/1.73 m^2^/year (*p* = 0.156).

### Comparison to UPCR-Matched Cohort

Of 74 eligible SKS patients with biopsy-proven IgAN who had not received immunosuppressive therapy, 16 were matched 1:1 to patients from the PP TRF budesonide cohort (*n* = 23). Pre-matched characteristics are shown in online supplementary Table S1 and online supplementary Figure 1; baseline characteristics of the matched cohorts are shown in Table 3.

**Table 3.** Baseline characteristics and outcome comparison between the TRF budesonide cohort and the UPCR-matched historical comparator cohort

Characteristic/Outcome	TRF budesonide (*n* = 16)	UPCR-matched SKS controls (*n* = 16)	*p* value
Baseline characteristics			
Age, years	41 (34 to 50)	57 (36 to 74)	0.052
Male sex, *n* (%)	8 (50.0)	12 (75.0)	0.273
White ethnicity, *n* (%)	12 (75.0)	15 (93.8)	0.333
Diabetes mellitus, *n* (%)	0 (0.0)	0 (0.0)	–
RAS inhibitor use at index date, *n* (%)	15 (93.8)	15 (93.8)	1.000
SGLT2 inhibitor use at any time during follow-up, *n* (%)^a^	14 (87.5)	7 (43.8)	0.023
Baseline UPCR, mg/mmol	149 (98 to 252)	152 (94 to 247)	1.000
Baseline eGFR, mL/min/1.73 m^2^	49 (34 to 70)	46 (34 to 70)	0.214
Time from biopsy to index date, months^b^	16.9 (3.1 to 26.0)	7.5 (3.2 to 29.7)	0.786
Outcome comparison			
Post-index eGFR slope, mL/min/1.73 m^2^/year	−2.06 (−5.11 to 0.98)	−2.90 (−5.00 to −0.30)	0.485

Values are median (IQR) unless otherwise stated. Continuous variables were compared using the Mann-Whitney U test and categorical variables using Fisher’s exact test, given the small cell counts. Controls were matched 1:1 to TRF budesonide recipients by nearest neighbour on baseline UPCR; because the propensity score was estimated from this single covariate, the procedure is equivalent to direct matching on baseline UPCR. The index date is treatment initiation for the TRF budesonide cohort and SKS enrolment for comparators.

SKS, Salford Kidney Study; RAS, renin-angiotensin system; SGLT2, sodium-glucose cotransporter-2; TRF, targeted-release formulation; UPCR, urine protein-to-creatinine ratio; eGFR, estimated glomerular filtration rate.

^a^ Exposure at any time during follow-up; at the index date, 14 of 16 TRF budesonide recipients and 3 of 16 matched comparators were established on an SGLT2 inhibitor. Matching on SGLT2 inhibitor use was not feasible because of near-complete non-overlap between cohorts (online suppl. Tables S3a, S3b). The net direction of the resulting bias cannot be determined; this comparison is contextual and does not support causal inference.

^b^ Biopsy dates were available for 11 of 16 comparator patients; the comparison is therefore between 16 and 11 patients.

Following matching, baseline UPCR was closely balanced between groups (149 vs. 152 mg/mmol). SMDs after matching indicated substantial residual imbalance in SGLT2 inhibitor use at index (1.90), age (−0.83), and baseline eGFR (0.55), against −0.07 for the matched variable, baseline UPCR (online suppl. Table S3a). SGLT2 inhibitor use at any point during follow-up remained higher in the TRF budesonide cohort (88% vs. 44%; *p* = 0.023), and the separation at the index date itself was greater still: only 3 of the 16 matched comparators (18.8%) were established on one at enrolment, and every candidate comparator receiving one had been enrolled in 2023 or 2024. In this set, the post-index eGFR slope was numerically less negative in the TRF budesonide cohort than in comparators (−2.06 vs. −2.90 mL/min/1.73 m^2^/year; *p* = 0.485).

Multivariable propensity score matching on age, sex, baseline eGFR and log baseline UPCR yielded 15 pairs with improved balance on all matched covariates (online suppl. Table S3c) and no evidence of a difference in post-index eGFR slope (median difference 1.17 mL/min/1.73 m^2^/year; *p* = 0.709; online suppl. Table S3d). Matching that additionally required exact agreement on SGLT2 inhibitor use at index was not estimable: only 4 of 74 comparator patients (5.4%) were receiving one at enrolment against 21 of 23 (91%) of the PP cohort, yielding at most a single pair (online suppl. Table S3b).

In a covariate-adjusted sensitivity analysis retaining all comparators with at least two post-index eGFR measurements on distinct dates (*n* = 61), there was no difference in eGFR slope after adjustment for age, baseline eGFR and log UPCR (+0.50 mL/min/1.73 m^2^/year, 95% CI: −4.22 to +5.22; *p* = 0.836). Addition of SGLT2 inhibitor use produced an unstable estimate reflecting near-collinearity with cohort membership rather than a treatment effect (online suppl. Table S4).

### Tolerability and Adverse Events

An adverse effect was recorded in 9 of 33 patients (27%). Treatment was discontinued in 7 (21%), in every case because of adverse effects: 6 had non-serious corticosteroid-related adverse effects, and 1 had a serious adverse event (staphylococcal septicaemia requiring hospital admission). Therapy was temporarily suspended and later resumed in 1 further patient, so that treatment was interrupted for any period in 8 of 33 (24%). No patient discontinued because of inefficacy. A full list of adverse effects is available in online supplementary Table S2.

## Discussion

In this single-centre real-world cohort of adults with IgAN, substantial residual proteinuria persisted despite 94% of patients receiving a RAS inhibitor and 91% an SGLT2 inhibitor. TRF budesonide was associated with a 57–60% reduction in UPCR at latest follow-up, additive to this optimised background therapy, and eGFR was stable across the treatment period and to latest follow-up. Neither the pre-to post-treatment slope comparison nor the comparison with a historical UPCR-matched cohort supports inference about eGFR trajectory: both are confounded by the timing and era of background SGLT2 inhibitor therapy. Discontinuation for adverse effects was more frequent than in the NefIgArd trial, though side effects were generally mild and in keeping with systemic glucocorticoid exposure; adverse effect frequency is not directly comparable given retrospective ascertainment.

Several targeted immune therapies have recently shown efficacy in IgAN [13–16], but access in the UK and Europe remains limited. Corticosteroids are therefore still the main licensed immune-modulating option, despite the considerable risk of serious adverse events [17, 18]. Supportive therapy with RAS and SGLT2 inhibition slows progression [19] but significant residual risk remains [20]. Our cohort illustrates this: despite 91% SGLT2 inhibitor use, median UPCR at treatment initiation exceeded 200 mg/mmol and 94% of patients remained above the KDIGO-recommended target of 50 mg/mmol [19]. Residual proteinuria is itself among the strongest predictors of progression, and in a cohort of this age (median 44 years) with established CKD (median eGFR 48 mL/min/1.73 m^2^), a sustained loss of even 2 mL/min/1.73 m^2^/year would bring the average individual close to kidney failure before their 60th birthday [20]. This provides a rationale for targeting the immunological driver of disease in such individuals [21]. NefIgArd established the efficacy of TRF budesonide, with a considerably better safety profile than oral corticosteroids. No participant, however, was receiving an SGLT2 inhibitor at randomisation, so its additive effectiveness on contemporary background therapy remains uncharacterised. Real-world series published to date report proteinuria reductions broadly comparable to ours [22], but are predominantly Chinese, largely confined to the 9-month treatment period, and do not describe background SGLT2 inhibitor use approaching that seen here.

The proteinuria reduction we observe at 9 months (41%) exceeds the 33.6% mean reduction from baseline in the NefIgArd TRF budesonide arm at the same timepoint, though the two are not strictly comparable. The trial’s concurrent placebo arm fell by 5.2% over the same interval, giving a placebo-adjusted reduction of 30.0% [7]; our analysis has no such subtraction and compares post-treatment UPCR with each patient’s own pre-treatment baseline. Because treatment initiation occurs at a point of peak or recently confirmed proteinuria, regression to the mean will contribute to the observed reduction independent of drug effect. Two features of our cohort may further amplify it: the predominance of active mesangial and segmental lesions with relatively limited chronic tubulointerstitial damage (M1 in 79%, S1 in 82%, T0 in 68%), consistent with selection for immunologically active disease, and treatment far earlier after biopsy than in the trial. On the other hand, prior corticosteroid exposure was more common here (21% against 8% in NefIgArd), which may reflect a more refractory phenotype, and baseline eGFR was lower (median 48 against 56 mL/min/1.73 m^2^). Chronic tubulointerstitial and sclerotic lesions are established predictors of adverse outcome in IgAN [23, 24] and are more prevalent at lower eGFR, so a cohort at this stage plausibly has less potential for functional response, though this cannot be demonstrated in these data. The PP estimate should not be read as evidence of incremental efficacy, since that cohort excludes early discontinuers by construction and characterises patients who tolerated and remained on therapy. The two estimates at latest follow-up are in fact indistinguishable (57% in the PP against 60% in the ITT cohort), so the selection inherent in the PP definition did not inflate the apparent response.

Two alternative explanations for the reduction in proteinuria are nonetheless unlikely to account for it. SGLT2 inhibitor therapy had been established for a median of 16.9 months (IQR 7.8–28.7) before budesonide initiation, and because its antiproteinuric effect is largely established within the first months of treatment, it is already reflected in the budesonide baseline in the great majority of patients. Regression to the mean is likewise implausible as a sole explanation for a median reduction of 60% sustained to a median of 11 months, particularly since the NefIgArd placebo arm, enrolled on the same basis of confirmed persistent proteinuria and therefore subject to the same regression pressure, fell by only 5.2% at 9 months [7].

The established relationship between UPCR and CKD progression in IgAN lends weight to the clinical relevance of the reductions observed here [25, 26]. Current KDIGO guidance recommends a proteinuria target of 0.5 g/day, corresponding to a UPCR of approximately 50 mg/mmol [19]. In our cohort, 7 of the 28 patients with a 9-month measurement (25%) reached it, and 9 of the full cohort of 33 (27%) at the latest follow-up, of whom 8 had started from a baseline UPCR of at least 100 mg/mmol. NefIgArd did not report attainment of an absolute proteinuria threshold, so no direct comparison with the trial is available. Attainment would in any case be expected to be lower here, given substantially higher baseline proteinuria than in the trial (median 212 against 145 mg/mmol). Relative change and absolute level answer different questions. Relative change is the better index of treatment response, and on that measure 25 of 33 patients (76%) achieved at least a 30% reduction in UPCR at latest follow-up and 21 (64%) at least a 50% reduction, though these describe within-patient change at a single timepoint and are not comparable with randomised response rates. Absolute level is the better index of residual risk, and on that measure 24 of 33 (73%) remained above 50 mg/mmol. In a UK registry cohort, approximately 30% of patients with time-averaged UPCR in the range immediately above this threshold developed kidney failure within 10 years [20]. Substantial residual risk therefore persists in most of this cohort despite a treatment response that would count as a success by relative criteria. That gap underlines the need for a wider range of immune-modulating options and for better ways to identify likely responders.

Whether this antiproteinuric effect translates into preserved kidney function cannot be determined from our data. eGFR was stable across the treatment period and to the latest follow-up. The post-treatment eGFR slope was numerically less steep than the pre-treatment slope, but this comparison is confounded by the timing of background therapy. SGLT2 inhibitor therapy was initiated within rather than before the pre-treatment observation window in 27 of the 28 patients with a useable start date, at a median of 1.5 years into that window. The pre-treatment window therefore contains the acute haemodynamic reduction in eGFR that follows SGLT2 inhibitor initiation, which steepens it, while the post-treatment window lies entirely within the period of established chronic benefit, which flattens it. Consistent with this, an exploratory within-patient analysis confined to the pre-treatment window showed a steeper slope after SGLT2 inhibitor initiation than before it, by a median of 2.40 mL/min/1.73 m^2^/year in the 19 patients in whom both windows were estimable. The initiation effect is unlikely to be the whole explanation: the steeper segment spans a median of 1.09 years, longer than the interval over which the acute haemodynamic reduction resolves, so a component of genuinely accelerating decline in the period preceding treatment is probable. That is itself expected in patients selected for TRF budesonide on the basis of perceived progression risk, and it means the pre-treatment slope reflects both a measurement artefact of background therapy and the selection that led to treatment. Neither is separable from the other in these data.

The segment preceding SGLT2 inhibitor initiation is the least contaminated by background therapy, at −1.79 mL/min/1.73 m^2^/year, but it is also the shortest, the furthest removed in time from the treatment decision, and estimated as a median of individual-level slopes rather than as a population-level fixed effect. It therefore cannot be interpreted against the post-treatment estimate of −0.96 mL/min/1.73 m^2^/year with useful precision. No attenuation of eGFR loss attributable to TRF budesonide is demonstrable in these data, and none of clinically relevant magnitude can be excluded. In NefIgArd, a difference in 2-year eGFR total slope of 2.78 mL/min/1.73 m^2^/year emerged over 2 years of follow-up [7], and our median post-treatment observation period of 11 months is likely insufficient for a comparable signal to accumulate.

The historical comparator analysis warrants particular caution and should be interpreted as descriptive rather than as evidence of treatment effect. Comparator patients were enrolled approximately a decade earlier and differ in age, baseline kidney function and background therapy, and the resulting imbalance in SGLT2 inhibitor exposure cannot be removed by matching or by adjustment within the available data. Matching on proteinuria also selected the less proteinuric half of the treated cohort (median baseline UPCR 149 against 220 mg/mmol in the PP cohort as a whole), so the matched comparison is not representative of the treated population. The net direction of residual bias cannot be determined: lower comparator SGLT2 inhibitor use would be expected to accelerate their decline, whereas comparators commencing one part-way through the interval over which their slope was estimated would have experienced attenuated decline. Indexing comparator follow-up to SKS enrolment rather than to biopsy positioned both groups at a similar point after diagnosis. It does not, however, make the two index points equivalent: comparators were selected by willingness and eligibility to enter a prospective cohort, and treated patients by clinician judgement of progression risk, so each group is selected on different criteria. More generally, the period over which SGLT2 inhibitors entered routine practice coincides with that from which any historical comparator must be drawn, so real-world evaluation of therapies introduced in this window will require contemporaneous, SGLT2 inhibitor-treated comparators rather than pre-post or historical designs.

The 21% adverse effect-related discontinuation rate in our cohort is higher than the 9% reported in NefIgArd. Our cohort was not subject to trial eligibility criteria excluding patients at elevated risk of corticosteroid-related harm, nor to the implicit selection for tolerability that accompanies trial recruitment. Concomitant CYP3A4 inhibitors, which increase systemic budesonide exposure, are also not systematically screened for in routine practice. Trial participants may be more likely to continue therapy through mild adverse effects, with dose interruption and active management available at scheduled visits. In routine care, the same symptoms more often result in permanent discontinuation. Adverse effects here were ascertained retrospectively from clinical correspondence and electronic records rather than by systematic symptom-directed screening. Passive ascertainment is expected to under-detect mild and self-limiting events while capturing events prompting discontinuation reliably, so the discontinuation rate is the more comparable measure and direct comparison of adverse event frequencies is not appropriate. That 7 of the 9 patients with any recorded adverse effect discontinued is itself consistent with under-ascertainment of milder events. The predominantly corticosteroid-associated nature of the events is consistent with systemic budesonide absorption, which can occur despite the targeted-release formulation [27, 28].

Several limitations warrant acknowledgement. The retrospective, single-centre design limits generalisability. At intermediate timepoints, patients who discontinued early contribute fewer observations, so those estimates are weighted towards patients continuing therapy and may overstate response; estimates at latest follow-up draw on the complete cohort of 33. The absence of a concurrent control group means the proteinuria reduction cannot be attributed to the drug in isolation: spontaneous proteinuria variability, regression to the mean and the independent antiproteinuric effect of SGLT2 inhibition could all contribute. Changes in other background therapy during follow-up were also not recorded. No patient received sparsentan before or during TRF budesonide treatment, as it became available at our centre only in November 2025, after the end of the treatment-initiation window; the combination warrants future evaluation [29]. The post-treatment observation period is too short to support conclusions about kidney protection, and the sample size provides limited power for slope comparison. Proteinuria was measured as a spot UPCR rather than a timed collection, and the absence of Gd-IgA1 or complement biomarker measurement precludes mechanistic interpretation [30]. The cohort is also too small to permit formal analysis of MEST-C components as predictors of treatment response, which will require larger multicentre series. Finally, eGFR was derived using the MDRD equation as reported by the hospital laboratory in both the treated and comparator cohorts, so absolute values are not directly comparable with trials reporting CKD-EPI-derived estimates, including NefIgArd.

## Conclusion

In a real-world IgAN cohort with near-universal RAS and SGLT2 inhibitor use, TRF budesonide was associated with a 57–60% reduction in proteinuria, additive to optimised supportive therapy, with eGFR stable across the treatment period and to latest follow-up. The attenuation of eGFR decline after treatment initiation did not reach significance and is confounded by SGLT2 inhibitor initiation within the pre-treatment observation window; the historical comparator analysis is contextual only. Neither supports inference about CKD progression. Discontinuation for adverse effects was more frequent than in NefIgArd, so real-world use warrants counselling on corticosteroid-related adverse effects before initiation and systematic symptom-directed screening at follow-up.

## Statement of Ethics

The evaluation of patients treated with targeted-release budesonide was conducted as a service evaluation in accordance with Northern Care Alliance NHS Foundation Trust governance policy (Research & Innovation reference: 25HIP90), for which formal research ethics committee approval and individual patient consent were not required. Comparator data were drawn from the Salford Kidney Study, a prospective observational cohort approved by the North West Research Ethics Committee (reference 15/NW/0818), in which all participants provided written informed consent. The study was conducted in accordance with the Declaration of Helsinki.

## Conflict of Interest Statement

This study was funded by an unrestricted grant from Stada, which markets targeted-release formulation budesonide in Europe. Jude Allen has received honoraria from Stada and Vifor. Smeeta Sinha has received honoraria from AstraZeneca, Boehringer Ingelheim, CSL Vifor, Novartis, GSK, Sanofi-Genzyme, Inozyme Pharma, Menarini, Amicus, Stada, Chiesi, Bayer and Santhera, and grant funding from AstraZeneca, Novartis, CSL Vifor, Johnson & Johnson and Sanofi-Genzyme. Smeeta Sinha was a member of the journal’s Editorial Board at the time of submission. Philip A Kalra receives grant funding from Medice, consulting fees from Boehringer Ingelheim and Stada, and honoraria from AstraZeneca, Pfizer, Pharmacosmos, Medice, GSK, Bayer, Boehringer Ingelheim and CSL Vifor. No other authors have any conflicts of interest to declare.

## Funding Sources

This study was funded by an unrestricted grant from Stada, which markets targeted-release formulation budesonide in Europe. The funder had no role in the design of the study, in the collection, analysis or interpretation of the data, or in the decision to submit the article for publication. The data were held and analysed by the investigators at the Northern Care Alliance NHS Foundation Trust. The funder did not review the manuscript before submission. The authors had full access to all of the data and take full responsibility for its integrity and for the decision to submit this manuscript.

## Author Contributions

T.M. and W.R.M.: conceptualisation, methodology, formal analysis, and writing (original draft). S.R.: data curation and writing (review and editing). J.A.: writing (review and editing). R.C.: data curation, project administration, supervision, and writing (review and editing). S.S.: and P.A.K.: supervision and writing (review and editing).

## Data Availability

The data that support the findings of this study are not publicly available in the interests of respecting participant privacy, but will be made available from the corresponding author upon reasonable request.
